# Urban-rural disparities in hypertension prevalence, detection, and medication use among Chinese Adults from 1993 to 2011

**DOI:** 10.1186/s12939-017-0545-7

**Published:** 2017-03-14

**Authors:** Jiajia Li, Leiyu Shi, Shixue Li, Lingzhong Xu, Wen Qin, Heng Wang

**Affiliations:** 1 Shandong University, Jinan, People’s Republic of China; 2 Johns Hopkins Bloomberg School of Public Health, Baltimore, USA; 3 Infirmary of Shandong University, Jinan, People’s Republic of China; 4 The Second Hospital of Shandong University, Jinan, People’s Republic of China

**Keywords:** Urban/rural, *hukou* system, Hypertension, Dynamic trends, China

## Abstract

**Background:**

China has experienced a rapid increase in hypertension over the past decade, especially in rural. Therefore, the aim of this research is to examine the dynamic trends in urban-rural disparities in hypertension prevalence, detection, and medication use among Chinese adults from 1993 to 2011.

**Methods:**

Data were extracted from the seven latest waves of the China Health and Nutrition Survey (CHNS). We used the *hukou* system to distinguish between urban and rural residents. Chi-square tests were performed to examine urban-rural gaps in hypertension prevalence, detection and medication use. Multiple logistic regressions were used to confirm these disparities and to explore whether the urban-rural gaps have narrowed or widened from 1993 to 2011, after controlling for health-related behaviors, BMI, demographic variables and socioeconomic characteristics. Blinder-Oaxaca decomposition technique was also used to calculate the extent to which urban-rural disparities reflect an endowments effect or a coefficients effect.

**Results:**

Hypertension prevalence, detection, and medication use among rural adults were significantly lower than urban adults, with the significant level at *p* < 0.001. The urban-rural gaps in hypertension prevalence and medication use gradually narrowed during the period 1993–2011, whereas the gaps in hypertension detection grew wider. After controlling for confounding variables, urban adults were about 24.5, 49.4, and 89.5% more likely to be hypertensive, detected, and medicated than their rural counterparts (*p* < 0.01), respectively. The Blinder-Oaxaca decomposition suggested that approximately 22 and 26% of the urban-rural gap in hypertension detection and medication use could be attributed to coefficient difference, respectively.

**Conclusions:**

Although hypertension prevalence among rural adults was comparable to that of urban adults, hypertension detection and medication use of rural adults were still suboptimal. Unusually large urban-rural gaps and an expanding trend in hypertension detection deserve the attention of health policymakers and researchers.

## Background

Hypertension, as a worldwide public health concern, has significant dose-response and predictive values to increased premature mortality [1]. A survey in 2005 showed that approximately 2.33 million cardiovascular deaths and 1.27 million premature cardiovascular deaths were attributable to hypertension in China [2]. In addition, China has experienced a rapid increase in hypertension over the past decade. The prevalence of hypertension among Chinese adults age 18 years and older has increased from 18.8% in 2002 to 25.2% in 2012 [3], and was reported to be as high as 29.6% in a survey in 2014 [4]. Controlling blood pressure for the 160 million hypertensive adults is considered as one of the most important priorities for chronic disease management in China [5].

Particular in rural China, the hypertension prevalence has dramatically increased from 20.4% in 1991 to 30.6% in 2007 [6], as a result of the rapid environmental and lifestyle changes that follow urbanization [5, 7]. Paradoxically, the rates of hypertension awareness, treatment, and control in rural China were frustratingly low. While China comprises one-fifth of the world’s population, more than half of the nation’s population lives in rural regions. High numbers of hypertension patients with poorly managed care of rural residents have brought about serious complications and become a massive burden on the health system in China [8].

In response, the Government of China views hypertension management and control as an important component of essential public health services offered through Community Health Service (CHS) organizations, as successful community-based interventions have been done [5]. Nevertheless, most of the CHS organizations are located in cities and urban areas. It is difficult for rural residents to obtain CHS chronic disease prevention, management, and control services. Previous studies have reported the urban-rural inequalities in healthcare use and health outcomes.

Moreover, different from many other parts of the world, China enforces a specific registration system to restrict internal migration, known as the *hukou* system. The *hukou* system, established in 1958, categorizes citizens into urban (non-agricultural) and rural (agricultural) residents of a given location [9–13]. Before the totally abolishment of the “agricultural” and “non-agricultural” *hukou* distinction in July 2014 in China, most of public service and welfare benefits are attached to a person’s *hukou* status, rather than the physical location, such as education system, health care, social security coverage, and so on [9] [11]. For example, China has established three basic social health insurance programs: Urban employee basic medical insurance (UEBMI) and Urban resident basic medical insurance (URBMI) in urban, as well as New cooperative medical system (NCMS) in rural. Both the URBMI and the NCMS are *hukou*-based social insurance program. That is, the rural *hukou* holders are stipulated to participate in the local NCMS [14, 15], while the URBMI only cover local *hukou* holders in urban [9, 16]. Although the UEBMI, which has highest benefits, is based on employment and not influenced by *hukou* status, only 10% of the rural *hukou* holders working in urban area are covered by the UEBMI [9]. This two-class health insurance system between urban and rural differ in health care coverage, protection, and reimbursement. Moreover, most high-quality health service facilities are located in urban, which are usually considered out-of-network or with low payment level and inconvenient procedures for NCMS enrollees [10, 16, 17]. All in all, *hukou* system has created a two-class society with sharp rural-urban distinctions [11]. Reforming or abolishing the *hukou* system was thought to be an integral part of any policy that deals with the growing rural–urban inequality [13]. Therefore, we use the *hukou* status rather than living location to distinguish between urban and rural in this study.

The main purpose of this study was to estimate the *hukou* distinguished urban-rural disparities in hypertension prevalence, detection and medication use. Also, we explored factors responsible for urban-rural disparities in hypertension prevalence, detection, and medication use, to draw public policy implications to further reform the health care and health insurance systems. Specially, given the health care system reforms initiated in the past few decades [18], we estimated the trends in urban-rural disparities by capitalizing on a longitudinal database from the China Health and Nutrition Survey (CHNS, to add up-to-date evidence to previous cross-sectional studies [4, 19–21].

## Methods

### Sampling

Data for this study were extracted from the China Health and Nutrition Survey (CHNS), an ongoing cohort project from 1989 beginning with eight provinces (Liaoning, Jiangsu, Shandong, Henan, Hubei, Hunan, Guangxi, Guizhou). A northeastern province (Heilongjiang) and three mega cities (Beijing, Shanghai, and Chongqing) have joined this cohort since 1997 and 2011, respectively. The twelve areas are representative and account for approximately half of China’s population. The CHNS has supplied nine rounds (1989, 1991, 1993, 1997, 2000, 2004, 2006, 2009, and 2011) data for public use until now. Taking into account that *hukou* status was added into questionnaires since 1993, we obtained eight waves of CHNS conducted between 1993 and 2011 in this study and focused on the hypertensive adults aged 18 years and above. The final study sample included 58,713 observations after excluding observations under 18 or with missing data.

### Measures/Variables

The main dependent variables of the present study were prevalence, detection, and medication use between urban and rural adults, which were assessed based on the US Seventh Joint National Committee report [22]. Prevalence was based on the question “Has a doctor ever told you that you suffer from high blood pressure?”, and was classified as yes if the respondent answered yes. Patients who had a SBP/DBP ≥ 140/90 mmHg were also classified as hypertensive patients. Both Systolic Blood Pressure (SBP) and Diastolic Blood Pressure (DBP) were measured by professional health workers in triplicate on the same day, which avoids recall bias associated with self-reported data. The average of the three measurements was used in the analysis to reduce the effect of measurement error [23–26]. Detection was measured by self-reported hypertension diagnoses and was classified as yes if a doctor diagnosed the respondent with high blood pressure. Medication use was measured by the question “Are you currently taking anti-hypertension drugs?” and was classified as yes for respondents whose answers were yes.

The key independent variable was whether a respondent belongs to an urban or rural registration (*hukou* dummy), which was based on the question “to which type of household registration do you belong.” Also, wave binary variables, together with an interaction term between *hukou* dummy and wave dummies, were added into the model to capture the period effects of hypertensive health care as well as dynamics trend of urban-rural disparities.

Given the socioeconomic variations between urban and rural, socioeconomic characteristics were controlled in the analyses. According to the previous studies [18, 20, 27–31], we also included other well-established risk factors including smoking habits, drinking frequencies, and BMI in the analyses, to capture the effect of lifestyle changes due to the urbanization and economic development. Demographic variables were taken into account to adjust the population heterogeneity between the two groups. Smoking habits was classified into never, ever, and current. Drinking frequencies was categorized as never, 3 drinks/month or less, 1–2 drinks/week, and at least 3 drinks/week. BMI was calculated by weight (kg) divided by square of height (m^2^), and coded into underweight (BMI < 18.5 kg/m^2^), normal (18.5 kg/m^2^ ≤ BMI < 24 kg/m^2^), overweight (24 kg/m^2^ ≤ BMI < 28 kg/m^2^), and obese (BMI > 28 kg/m^2^) using the World Health Organization criteria. Demographic variables included age (years), sex (male/female), marital status (married/others). Socioeconomic characteristics included education (formal education years in a regular school), household income per capita (RMB in 2011 value), types of medical insurance (classified into none, NCMS, URBMI,, and others-including commercial medical insurance, government free medical insurance), and area (categorized as Western- Guanxi, Guizhou, Chongqing; northeastern- Liaoning, Heilongjiang; central- Henan, Hubei, Hunan; eastern- Jiangsu, Shandong, Beijing, Shanghai).

### Statistical analysis

Data analyses were conducted by using the STATA 14.0 and carried out by descriptive statistics, logistic regressions, Blinder-Oaxaca decomposition techniques.

Descriptive statistics for hypertension prevalence, detection, and medication use among urban and rural adults were reported as proportions, with corresponding chi-square and the *p*-values to examine whether there were statistically significant differences between urban and rural adults. Demographic and socioeconomic statuses were also estimated as means for continuous variables and proportions for categorical variables. We conducted chi-square tests for dichotomous variables and t-tests for continuous variables and reported their *p*-values.

We adopted logistic regression method using the pooled cross-sectional data to investigate the urban-rural disparities in multivariate analyses adjusted for confounding variables. Adjusted odds ratios with their *p*-values were reported. The model was specified as:

1 $$ In\left(\frac{P_i}{1-{P}_i}\right)={\beta}_0+{\beta}_1 HUKO{U}_i+{\beta}_2 w a v{e}_i+{\beta}_3\left( HUKO{U}_i\ast wav{e}_i\right)+{\displaystyle {\sum}_1^n{\alpha}_n{X}_{n i}} $$

Where *P*_*i*_ represented the probability of hypertension prevalence, detection, and medication use; *HUKOU*_*i*_ indicated whether the respondent *i*’s *hukou* was urban or rural; *wave*_*i*_ represented the time dummies to explore the dynamic evolution from 1993 to 2011; *X*_*ni*_ were control variables. Coefficients *β*_1_, *β*_2_, and *β*_3_ represented urban-rural disparities, time trends, and time trends of urban-rural disparities, respectively.

The Blinder-Oaxaca decomposition [32, 33] was a counterfactual method with an assumption that “what the probability of hypertension prevalence, detection, and medication use would be if rural adults had the same characteristics as their urban counterparts?”. Based on it, we divided the model indicating urban-rural disparities into two parts by using the Blinder-Oaxaca decomposition as followed:

2 $$ E\left({P}_u-{P}_r\right)= E{\left[{Z}_u\right]}^{\prime}\left({\beta}_u-{\beta}_r\right)+{\left( E\left[{Z}_u\right]- E\left[{Z}_r\right]\right)}^{\prime }{\beta}_r $$

Where u represented urban adults and r represented rural adults; Z represented all the independent variables appeared in Eq. (1); β represented the estimated coefficients. The second term in Eq. (2) traced the differentials from the magnitude of the variables controlled in the regression, it indicated “endowments effect”(or “explained component”); while the first term was “coefficients effect” (or “unexplained component”) traces the differences that are attributable to the effect of the variables. Decomposing urban-rural hypertension differences into endowments and coefficients effects has strong policy implications since the evidence of coefficients effect would reflect that urban and rural hukou holders endowed with the same characteristics do not enjoy the same level of hypertension prevalence, detection, and medication use.

## Results

### Descriptive results

Table 1 displays descriptive statistics for the variables used in this study for the entire sample as well as the rural and urban samples. Rural hypertensive adults accounted for approximately 59.72% of the sample. The proportion of hypertension prevalence, detection, and medication use among rural adults were significantly lower than urban adults, with significant level at *p* < 0.001. Descriptive results indicate that urban and rural adults were also significantly (*p* < 0.001) different in all the control variables, especially in socioeconomic status. The mean value of household income per capital of urban adults is nearly twice of rural income. Meanwhile, rural adults were more likely to be uninsured and have less regular schooling years than their urban counterparts. Moreover, compared with urban adults, rural adults are more likely to be underweight, but less likely to be overweight and obese.

**Table 1:** Descriptive statistics

Variables	All	Rural	Urban	*p* ^a^
Prevalence, n (%)				0.000
No	44,614 (75.99)	27,473 (78.35)	17,141 (72.49)	
Yes	14,099 (24.01)	7593 (21.65)	6506 (27.51)	
Detection, n (%)				0.000
No	8525 (60.47)	5236 (68.96)	3289 (50.55)	
Yes	5574 (39.53)	2357 (31.04)	3217 (49.45)	
Medication Use, n (%)				0.000
No	1289 (23.13)	731 (31.01)	558 (17.35)	
Yes	4285 (76.87)	1626 (68.99)	2659 (82.65)	
Control variables				
Age, mean (SD)	47.59 (15.62)	46.57 (15.20)	49.11 (16.12)	0.000
Sex, n (%)				0.000
Male (Ref.)	27,591 (46.99)	16,174 (46.12)	11,417 (48.28)	
Female	31,122 (53.01)	18,892 (53.88)	12,230 (51.72)	
Marital status, n (%)				0.000
Married (Ref.)	48,275 (82.22)	29,244 (83.40)	19,031 (80.48)	
Others	10,438 (17.78)	5822 (16.60)	4616 (19.52)	
Education, mean (SD)	7.13 (4.33)	5.92 (3.83)	8.93 (4.40)	0.000
Income (RMB in 2011 value), mean (SD)	8765.63 (12149.52)	6555.21 (9827.00)	12043.44 (14328.87)	0.000
Types of medical insurance, n (%)				0.000
None (Ref.)	29,488 (50.22)	19,956 (56.91)	9532 (40.31)	
NCMS	15,056 (25.64)	13,728 (39.15)	1328 (5.62)	
URBMI	2707 (4.61)	233 (0.66)	2474 (10.46)	
UEBMI	4728 (8.05)	251 (0.72)	4477 (18.93)	
Others	6734 (11.47)	898 (2.56)	5836 (24.68)	
Smoking, n (%)				0.000
Never	40,235 (68.53)	23,635 (67.40)	16,600 (70.20)	
Ever	1487 (2.53)	706 (2.01)	781 (3.30)	
Current	16,991 (28.94)	10,725 (30.59)	6266 (26.50)	
Drinking, n (%)				0.000
Never (Ref.)	38,910 (66.27)	23,476 (66.95)	15,434 (65.27)	
≤ 3 drinks/month	6272 (10.68)	3515 (10.02)	2757 (11.66)	
1–2 drink/week	4863 (8.28)	2804 (8.00)	2059 (8.71)	
≥ 3 drinks/week	8668 (14.76)	5271 (15.03)	3397 (14.37)	
BMI, n (%)				0.000
Underweight (Ref.)	3917 (6.67)	2646 (7.55)	1271 (5.37)	
Normal	34,384 (58.56)	21,889 (62.42)	12,495 (52.84)	
Overweight	15,730 (26.79)	8267 (23.58)	7463 (31.56)	
Obese	4682 (7.97)	2264 (6.46)	2418 (10.23)	
Area, n (%)				0.000
Western (Ref.)	15,065 (25.66)	10,112 (28.84)	4953 (20.95)	
Northeastern	18,208 (31.01)	11,578 (33.02)	6630 (28.04)	
Central	10,745 (18.30)	6360 (18.14)	4385 (18.54)	
Eastern	14,695 (25.03)	7016 (20.01)	7679 (32.47)	
Wave, n (%)				0.000
1993 (Ref.)	6644 (11.32)	4287 (12.23)	2357 (9.97)	
1997	7347 (12.51)	4660 (13.29)	2687 (11.36)	
2000	7704 (13.12)	4618 (13.17)	3086 (13.05)	
2004	7952 (13.54)	4917 (14.02))	3035 (12.83)	
2006	7425 (12.65)	5007 (14.28)	2418 (10.23)	
2009	9303 (15.84)	5498 (15.68)	3805 (16.09)	
2011	12,338 (21.01)	6079 (17.34)	6259 (26.47)	
observations	58,713	35,066 (59.72)	23,647 (40.28)	

^a^ χ^2^ tests for dichotomous variables and t-tests for continuous variables

**Fig. 1:**
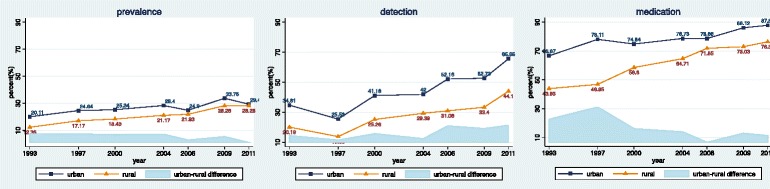
Hypertension prevalence, detection and medication use among urban and rural adults in China (1993–2011)

Figure 1 shows urban-rural disparities as well as the trends in hypertension prevalence, detection, and medication use from 1993 to 2011. As shown in the figure, although hypertension prevalence, detection, and medication use elevated during 1993 to 2011 for both urban and rural adults, the urban-rural gaps still persistented. It is evident that urban-rural disparities of hypertension detection and medication use were considerably larger than hypertension prevalence. However, the gap gradually narrowed for hypertension prevalence and medication use over time from 1993 to 2011, whereas extended for hypertension detection.

### Logistic regression analyses

Table 2 presents the results of logistic regression analyses for urban-rural disparities in hypertension prevalence, detection, and medication use. After controlling for confounding variables, urban adults were about 24.5% more likely to be hypertensive (OR = 1.245; *p* < 0.01) than rural adults. Similar significant differences were observed in detection and medication use: rural hypertensive adults were about 49.4% less likely to be detected (OR = 1.494; *p* < 0.01) and 89.5% less likely to be medicated (OR = 1.895; *p* < 0.01) than their urban counterparts, respectively.

**Table 2:** Logistic regressions reporting urban-rural disparities of hypertension prevalence, detection, and medication use

Variables	Prevalence Odds ratio	Detection Odds ratio	Medication Use Odds ratio
*hukou*: Urban	1.245***	1.494***	1.895**
	(0.099)	(0.232)	(0.536)
Types of medical insurance: NCMS	1.008	0.880*	0.924
	(0.041)	(0.062)	(0.123)
Types of medical insurance: URBMI	1.096	1.117	1.521**
	(0.073)	(0.117)	(0.301)
Types of medical insurance: UEBMI	1.139**	1.259**	1.455**
	(0.069)	(0.121)	(0.255)
Types of medical insurance: others	1.069	1.209***	1.106
	(0.044)	(0.082)	(0.133)
Age	1.065***	1.038***	1.033***
	(0.0011)	(0.002)	(0.004)
Sex: male	0.735***	1.469***	0.983
	(0.023)	(0.077)	(0.095)
Marital status: married	1.077**	0.803***	0.947
	(0.034)	(0.042)	(0.095)
Education	0.983***	1.024***	1.014
	(0.003)	(0.006)	(0.010)
Income	1.000	1.000***	1.000***
(RMB in 2011 value)	(0.000)	(0.000)	(0.000)
Smoking: ever	1.089	1.582***	0.779
	(0.071)	(0.150)	(0.121)
Smoking: current	1.022	0.928	0.899
	(0.032)	(0.048)	(0.085)
Drinking: ≤3 drinks/month	1.020	0.859**	0.670***
	(0.040)	(0.061)	(0.086)
Drinking: 1–2 drink/week	1.024	0.755***	0.567***
	(0.045)	(0.062)	(0.086)
Drinking: ≥3 drinks/week	1.194***	0.848***	0.524***
	(0.041)	(0.050)	(0.056)
BMI: normal	1.673***	1.423***	1.566**
	(0.090)	(0.150)	(0.285)
BMI: overweight	3.475***	2.021***	1.890***
	(0.193)	(0.217)	(0.350)
BMI: obese	7.405***	2.593***	2.601***
	(0.460)	(0.293)	(0.509)
Area: central	1.465***	1.185***	1.322***
	(0.045)	(0.067)	(0.133)
Area: northeastern	2.012***	1.092	0.939
	(0.071)	(0.069)	(0.103)
Area: eastern	1.577***	1.397***	1.461***
	(0.052)	(0.080)	(0.150)
Wave: 1997	1.285***	0.596***	1.030
	(0.085)	(0.094)	(0.311)
Wave: 2000	1.184***	1.137	1.637*
	(0.077)	(0.161)	(0.429)
Wave: 2004	1.154**	1.330**	2.013***
	(0.074)	(0.181)	(0.505)
Wave: 2006	1.082	1.398**	2.783***
	(0.072)	(0.194)	(0.728)
Wave: 2009	1.491***	1.583***	2.859***
	(0.107)	(0.228)	(0.773)
Wave: 2011	1.277***	2.266***	3.175***
	(0.091)	(0.323)	(0.843)
Urban*1997	0.904	0.985	1.611
	(0.092)	(0.205)	(0.636)
Urban*2000	0.857	1.018	0.872
	(0.085)	(0.192)	(0.296)
Urban*2004	0.908	0.810	0.796
	(0.089)	(0.148)	(0.264)
Urban*2006	0.766***	1.191	0.553*
	(0.079)	(0.226)	(0.191)
Urban*2009	0.741***	0.923	0.706
	(0.074)	(0.170)	(0.237)
Urban*2011	0.688***	0.996	0.662
	(0.068)	(0.181)	(0.216)
Constant	0.004***	0.015***	0.074***
	(0.000)	(0.000)	(0.028)
Observations	58,713	14,099	5574

Robust standard errors are reported in parenthesis. *** *p* < 0.01, ** *p* < 0.05, * *p* < 0.1

Changes in hypertension prevalence, detection, and medication use from 1993 to 2011, after controlling for other factors, also could be discovered in Table 2. There was a statistically significant upward trend of hypertension detection from the year 2004. For example, the probability of detection in 2011 is 2.266 times that of 1993. Similarly, we noticed a statistically significant trend of increased hypertension medication use from the year 2000. However, we could not find a period effect in prevalence after the confounding variables were accounted for.

To further clarify the time trend on urban-rural disparities, the interaction effect between *hukou* dummy and wave dummies could be found in Table 2. Urban-rural difference in hypertension prevalence significantly decreased from 2006. The gap in 2011 was about 30% narrower than 1993. A similar declining trend in the urban-rural difference of medication use could be found from 1997 but was insignificant. No apparent narrowing or extended trends can be found in urban-rural detection gap.

Consistent with these findings, adults with any health insurance were more likely to be hypertensive than adults who were not covered by any health insurance, especially for adults with UEBMI (OR = 1.139; *p* < 0.05). None-insured adults had a lower probability of detection compared with UEBMI (OR = 1.259; *p* < 0.05) and other insurance participants (OR = 1.209; *p* < 0.01), but had a higher probability compared with NCMS participants (OR = 0.880; *p* < 0. 1).

We can observe a distinct regional effect in logistic regression results. Adults in northeastern provinces had the greatest probability of being hypertensive, but the worst probability of medication use. At the same time, eastern adults had the highest qualities of detection and medication use amongst hypertensives compared with other regions. In contrast, western adults’ probability of prevalence, detection, and medication use was significantly lower than the other areas.

### Decomposition analyses

We provide the Blinder-Oaxaca decomposition results in Table 3. The results show that the probabilities of being hypertensive were 27.51% for urban adults and 21.65% for rural adults. Only the endowments effect was significant and could explain 5.55% of the total 5.86% urban-rural difference in hypertension prevalence. Results for detection and medication use were somewhat different. The overall urban-rural differences in the predicted probability of detection and medication use were 18.40% and 13.67%, respectively. Both endowments effect and coefficients effect were significant in logistic decompositions for detection and medication use, though the former was more important. About 4.20% of the total urban-rural difference in hypertension detection could be attributed to coefficients effect. There were comparable findings for medication use. The total differences and difference attributed to coefficients effect between urban and rural hypertension detection and medication use were greater than that of hypertension prevalence.

**Table 3:** Blinder-Oaxaca decomposition results between urban and rural adults

	Prevalence	Detection	Medication Use
Predicted probability			
Urban	27.51%^***^	49.45%^***^	82.65%^***^
	(0.0029)	(0.0062)	(0.0066)
Rural	21.65%^***^	31.04%^***^	68.99%^***^
	(0.0022)	(0.0053)	(0.0095)
Difference in predicted probability			
Total difference (urban-rural)	5.86%^***^	18.40%^***^	13.67%^***^
	(0.0036)	(0.0082)	(0.0116)
Difference due to endowments effect	5.55%^***^	14.21%^***^	10.06%^***^
	(0.0027)	(0.0060)	(0.0082)
Difference due to coefficients effect	0.31%	4.20%^***^	3.61%^***^
	(0.0024)	(0.0056)	(0.0082)

Robust standard errors are reported in parenthesis. *** *p* < 0.01, ** *p* < 0.05, * *p* < 0.1

## Discussion

The present study showed that rural hypertensive adults had lower prevalence, detection, and medication use than their urban counterparts, which was consistent with earlier findings [7, 19, 20, 23]. These differences were apparent for the proportion as well as the probability after other factors were accounted for. Controlling for the confounding factors, we found that the net advantages of urban *hukou* on hypertension prevalence, detection, and medication use were 1.245, 1.494 and 1.895, respectively.

We also concluded that the probability of prevalence disparities between urban and rural significantly declined from 2004 to 2011 while holding everything else constant. Unfortunately, this appeared to reflect, not a decrease in urban prevalence, but relatively more dramatic improvement in rural locales. The raw proportion of rural hypertension prevalence showed a persistent upward trend, while two decreases of urban prevalence occurred in 2006 and 2011. These inconsistent trends between probability and raw proportion of urban-rural gap reflected the urban-rural discrepancies in covariates, such as BMI and age. Consistently, the results of Blinder-Oaxaca decomposition showed that the endowments effect, rather than the coefficients effect, accounted for most of the urban-rural difference in hypertension prevalence. In other words, lifestyle and dietary pattern change, as a consequence of urbanization and economic growth, may have contributed to the relatively rapid increase in hypertension prevalence among rural adults. Rapidly expanding urbanization has spilled over in China from the 1980s. In particular, the rate of urbanization has strikingly increased from 35.39% in 2000 to 51.27% in 2011, with a great acceleration compared to ever before. As the occupation of agricultural land expanded, farmers began to engage in sedentary lifestyle instead of manual labor [34], and the amount of physical activities of rural residents was significantly reduced [35]. Almost during the same period, income growth increased the intake of higher calorific value and higher fat food among rural adults [35, 36]. The increased prevalence of hypertension in rural not only presents a formidable public health challenge but also may speed up the health expenditure rise. As rural residents would face with heavier hypertension care burden because of lower income and lower insurance benefit [37], what we should focus on next is to make hypertension more preventable and affordable.

Furthermore, we found that the raw urban-rural gap in hypertension detection not only persisted but also extended over the time from 1993 to 2011. Take the detection rate of 2011 as an example. The detection rate of urban adults was 65.6%, comparable to 66.5% reported in American National Health and Nutrition Examination Survey 2003–2004 [30]. Detection of rural adults was only 44.10% and was suboptimal. Indeed, the decomposition results suggested that about 4.20% of the total 18.4% urban-rural difference in hypertension detection could be attributed to the coefficients effect, which also explained the insignificant trend of urban-rural disparities after adjustment by covariates. The “coefficients effect”, which was considered as discrimination in previous studies [32, 33, 38], could reflects some hukou-related institutional difference and unobserved factors here. First, due to the unbalanced distribution of educational resources between urban and rural, rural residents had a lower degree of education than their urban counterparts, which might explain their lack of knowledge or awareness of hypertension [27]. Second, most of the health improvement activities, such as hypertension screening and management programs, were held in urban areas and covered by urban insurance, which caused a barrier to equal access for rural insurance enrollees [10]. Apart from this, the Blinder-Oaxaca decomposition showed that both the overall disparity and coefficients effect in hypertension detection were more critical than those in hypertension prevalence and medication use. Thus, the most important priority in rural China is to improve early detection of hypertension. Accordingly, more primary care institution targeting hypertension awareness should be scaled up to pursue better access to hypertension education and hypertension screening for rural residents. Besides the increasing health facilities for rural residents, enhanced efforts toward the solution of human resource shortage should be implemented in China. Remarkable increases of hypertension detection have been apparent for both urban and rural residents from 2009 to 2011, which reflected the effect of the Basic Public Health Services program initiated since 2009 [18, 35]. We can also expect the effect on rural hypertension as the program further spread in rural.

An encouraging finding was that the urban-rural gap in hypertension medication use had been narrowed since 2000, though not significantly. The narrowing probably reflected the improvement of income growth in rural, as well as the ongoing reform of the rural health system and *hukou* system. For example, the Central Government of China released six NO.1 Central Documents from 2003 to 2009, targeting the strengthening of the basic rural health care infrastructure, establishment of rural health insurance system, and income improvement in rural area [37]. Accordingly, US$1.4 million financial support was invested in establishing rural health service system between 2004 to 2007 [37]. In 2009, China introduced a nationwide health care system reform and ramped up investment in public health and insurance. In addition, the development and the continuous improvement of the NCMS also played an active role in the gap narrowing [15, 39]. With regard to the *hukou* system, the conversion from rural to urban *hukou* (called “nongzhuanfei” in Chinese) has become less difficult since 2000 [9]. Meanwhile, the *hukou* restriction in insurance enrollment has become more and more relaxed, which may make advance in lower out-of-pocket and hypertension treatment promotion.

Distinct insurance difference and regional effect could be observed in our research. Urban insurance enrollees had higher detection and medication use than rural insurance enrollees and uninsured adults. Therefore, further efforts are needed to integrate the decentralized insurance system into one uniform system. Fortunately, several regions in China, such as Tianjin, Jiangsu, Shandong, have recently carried out this type of trial aimed to offer the same health insurance coverage for both rural and urban population. Furthermore, adults in northeastern and western provinces had worse detection and medication use than other regions, which should assign sufficient priorities and resources to the western and northeastern area.

Consistent with previous studies [4, 20, 23, 27], age and BMI, two well-known associated factors, had positive effects on hypertension prevalence, detection, and medication use. As revealed in this study, adults with better education had a lower probability to suffer from hypertension and had higher detection and medication use, which indicate the importance of health education. An interesting finding was that the heavier a drinker was, the higher prevalence and the lower detection and medication use he/she had. Therefore, unhealthy lifestyle not only means higher risk but also means less emphasis on health, which suggested extra attention intervene these health hazard behaviors.

## Conclusions

Although we observed higher hypertension prevalence of urban adults, the comparable level of rural adults suggests that hypertension of rural residents is in need of attention. Moreover, significant urban-rural gaps were observed in hypertension detection on the basis of CHNS from 1993 to 2011. Hypertension prevalence of rural may extend to that of urban in the future if action is not taken now. Looking to the future, we recommend that four efforts should be made to prevent and aware hypertension aimed at rural residents. First, improve health education for rural residents to promote a healthy lifestyle and hypertension prevention. Second, expand medical insurance for hypertension to improve the affordability. Third, strengthen primary care to rural residents, such as regular hypertension screening and monitoring, to timely detect hypertension. Fourth, carry out community hypertension intervention based on the experience of the urban. Accordingly, China should make extraordinary efforts to improve the quantity and efficiency of rural primary care services. Moreover, although the *hukou* institution was abolished in 2014, findings of the present study provides evidence for health policy makers of the striking necessity for the abolishment.

### Limitations

This study has some limitations that must be acknowledged. First, due to data limitations, we used anti-hypertensive medication use to represent hypertension medication use. Although there are many other methodologies including the popular salinity control strategy to treat hypertension in China, the questionnaire only involves the issue of whether the respondents are currently taking anti-hypertensive drugs [40]. Second, the CHNS is a pooled cross-sectional dataset, which may contribute to sample bias. On one hand, some patients in the CHNS were repeatedly measured across different years and might have better awareness than the general population in China [23]. On the other hand, respondents in each year vary in age, education, and health-related behaviors, which may result in inaccurate results of the trends, although we have controlled these factors to adjust for this bias in regression analyses.

## Abbreviations

CHNS: China Health and Nutrition Survey
CHS: Community Health Service
DBP: Diastolic Blood Pressure
NCMS: New cooperative medical system in rural
SBP: Systolic Blood Pressure
UEBMI: Urban employee basic medical insurance
URBMI: Urban resident basic medical insurance
